# Predicting Time in Range Without Hypoglycaemia Using a Risk Calculator for Intermittently Scanned CGM in Type 1 Diabetes

**DOI:** 10.1002/edm2.70020

**Published:** 2024-12-24

**Authors:** Fernando Sebastian‐Valles, Jose Alfonso Arranz Martin, Julia Martínez‐Alfonso, Jessica Jiménez‐Díaz, Iñigo Hernando Alday, Victor Navas‐Moreno, Teresa Armenta Joya, Maria del Mar del Fandiño García, Gisela Liz Román Gómez, Jon Garai Hierro, Luis Eduardo Lander Lobariñas, Carmen González‐Ávila, Purificación de Martinez de Icaya, Vicente Martínez‐Vizcaíno, Miguel Antonio Sampedro‐Nuñez, Mónica Marazuela

**Affiliations:** ^1^ Universidad Autónoma de Madrid, Department of Endocrinology and Nutrition Hospital Universitario de La Princesa, Instituto de Investigación Sanitaria de La Princesa Madrid Spain; ^2^ Department of Family and Community Medicine Hospital La Princesa/Centro de Salud Daroca Madrid Spain; ^3^ Department of Endocrinology and Nutrition Hospital Universitario Severo Ochoa Leganés Spain; ^4^ Department of Endocrinology and Nutrition Hospital Universitario Basurto Bilbao Spain; ^5^ Department of Neurology Hospital Universitario Infanta Elena Valdemoro Spain; ^6^ Health and Social Care Research Center Universidad de Castilla‐La Mancha Cuenca Spain; ^7^ Facultad de Ciencias de la Salud Universidad Autónoma de Chile Talca Chile

**Keywords:** continuous glucose monitoring systems, intermittently scanned continuous glucose monitoring, optimal control, predictive models, type 1 diabetes

## Abstract

**Purpose:**

To investigate the impact of clinical and socio‐economic factors on glycaemic control and construct statistical models to predict optimal glycaemic control (OGC) after implementing intermittently scanned continuous glucose monitoring (isCGM) systems.

**Methods:**

This retrospective study included 1072 type 1 diabetes patients (49.0% female) from three centres using isCGM systems. Clinical data and net income from the census tract were collected for each individual. OGC was defined as time in range > 70%, with time below 70 mg/dL < 4%. The sample was randomly split in two equal parts. Logistic regression models to predict OGC were developed in one of the samples, and the best model was selected using the Akaike information criterion and adjusted for Pearson's and Hosmer–Lemeshow's statistics. Model reliability was assessed via external validation in the second sample and internal validation using bootstrap resampling.

**Results:**

Out of 2314 models explored, the most effective predictor model included annual net income per person, sex, age, diabetes duration, pre‐isCGM HbA1c, insulin dose/kg, and the interaction between sex and HbA1c. When applied to the validation cohort, this model demonstrated 72.6% specificity, 67.3% sensitivity, and an area under the curve (AUC) of 0.736. The AUC through bootstrap resampling was 0.756. Overall, the model's validity in the external cohort was 80.4%.

**Conclusions:**

Clinical and socio‐economic factors significantly influence OGC in type 1 diabetes. The application of statistical models offers a reliable means of predicting the likelihood of achieving OGC following isCGM system implementation.

## Graphical abstract

Risk calculator predicts optimal glycemic control (Time in range >70%, Time below range <4%) based on income, HbA1c, gender, insulin dose, and age after isCGM placement.

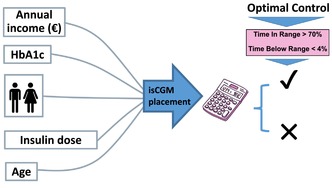

## 1 Introduction

Robust evidence accumulated over the years supports that strict glycaemic control can mitigate the adverse effects of hyperglycaemia in patients with type 1 diabetes (T1D) [1]. Self‐assessment of capillary glucose levels through glucose monitoring marked the outset of improved chronic control of T1D [2]. Over the past decade, the advent of continuous and intermittently scanned continuous glucose monitoring (CGM and isCGM) devices has been associated with reduction in glycated haemoglobin (HbA1c) levels in both children and adults with insulin‐treated diabetes [3, 4]. Furthermore, highly diverse real‐world studies have demonstrated the success of the widespread use of these devices in improving glycaemic control [5].

CGM and isCGM sensors offer a spectrum of glucose metrics whose significance in evaluating the glycaemic condition of individuals with diabetes is endorsed by international consensus guidelines [6]. These glucose metrics correlate with HbA1c levels [7] and, in some cases, offer even more informative insights [8]. Presently, prospective clinical studies employ CGM devices to collect complementary data in therapeutic interventions in T1D [6]. One of the glucose metrics provided by CGM systems, Time in Range (TIR) between 70 and 180 mg/dL, is widely used as a primary outcome measure in T1D studies. Then, 5% changes in TIR are considered clinically significant [9] and TIR > 70% is regarded as indication of HbA1c target achievement [10].

On the other hand, severe hypoglycaemia cannot be assessed using CGM metrics. In this case, time below range < 70 mg/dL (TBR) is proposed as the best predictor of severe hypoglycaemia [11], and the currently recommended threshold is established at TBR < 4% [6]. Since hypoglycaemia is a limiting factor in glycaemic management, studies addressing the prediction of hypoglycaemic events are emerging [12]. However, predictive models assessing, at the time of CGM sensor placement, the likelihood of achieving the recommended optimal glycaemic control (TIR > 70% and TBR < 4%) remain to be developed.

In this context, the aim of this study is to develop predictive models for optimal glycaemic control based on clinical and socio‐economic variables influencing T1D chronic control [13, 14]. These models could predict of the probability of achieving the recommended glycaemic control with an isCGM sensor during clinical follow‐up.

## 2 Materials and Methods

This follow‐up study included 1255 individuals attending three Spanish hospitals (Hospital Universitario de Basurto, Hospital Universitario de la Princesa and Hospital Universitario Severo Ochoa), located in three different geographical areas. All the participants were regular users of isCGM (FreeStyle Libre, Abbott), with a mean usage duration of 2.3 ± 1.4 years. Glucose metrics were collected from cloud downloads on the Libreview platform over a 14‐day period in November 2022.

Inclusion criterion was T1D diagnosis. Exclusion criteria were patients with a diagnosis of type 2, Maturity onset diabetes of the young, or other types of diabetes, those with a usage time < 70%, as recommended by scientific evidence [6, 15], and those who did not have a download of sensor data within 30 days prior to data collection. This study followed the “Strengthening the Reporting of Observational Studies in Epidemiology” guidelines [16]. Ethical approval was obtained from the Research Ethics Committee of Hospital de La Princesa, Madrid (Study number: 5084‐01/2023), and the research was conducted in accordance with the principles of the Declaration of Helsinki.

### 2.1 Procedures

Before using the FreeStyle monitor, all patients underwent a training session in accordance with international recommendations [6]. The system comprises a subcutaneously implanted glucose oxidase‐based electrochemical sensor, which is replaced every 14 days. This sensor transmits interstitial glucose measurements wirelessly to a receiver, and these data are securely stored in the cloud via the Libreview platform. Written instructions were provided to all patients on utilising the isCGM data for real‐time insulin dose adjustments and on using the Libreview cloud platform for retrospective review of glucose data to inform future insulin dose adjustments. All patients were advised on how to adapt their insulin dosages and hypoglycaemia management based on their individual glucose profiles and trends.

### 2.2 Data Collection

Glucose metrics were obtained at 14‐day intervals from the Libreview platform using the FreeStyle 2 device (FreeStyle Libre 2, Abbott). The collected variables included TIR, TBR for glycaemia < 70 mg/dL, and time above range for glycaemia > 180 mg/dL, number of daily readings, sensor usage, coefficient of variation (CV), and standard deviation (SD).

Optimal glycaemic control was defined when as achievement of a TIR > 70% and a TBR < 4%, according to recommended criteria [6]. Additionally, clinical data, laboratory test results, and information on pharmacologic treatment for T1D were extracted from electronic health records. These data encompassed sex, age, duration of diabetes mellitus, diabetes type, body mass index (BMI), smoking habits, use of continuous subcutaneous insulin infusion (CSII), HbA1c levels at the time of CGM sensor placement, CGM usage duration, age at disease onset, insulin dosage, nephropathy, and retinopathy. The classification of both complications followed international standards [17, 18]. Glycated haemoglobin was routinely assessed using liquid chromatography (ADAMS A1c HA8180 V ARKRAY).

As a marker of socio‐economic status (SES), we used the average annual net income per person of the census tract for each participant, which is published periodically by the National Statistics Institute (Atlas de Distribución de Renta de los Hogares 2020. Retrieved from https://www.ine.es/componentes_inebase/ADRH_total_nacional.htm Accessed 12 October 2023), as was used in the past [19, 20].

### 2.3 Statistical Analysis

Categorical variables were expressed as percentages and absolute values, while continuous variables were presented as mean ± SD, median and interquartile range, or range, depending on the Gaussian distribution of the data. The Kolmogorov–Smirnov test and graphical methods were employed to assess the normality of continuous variables. Differences between variables were considered statistically significant at *p* < 0.05. All statistical analyses were performed using STATA 17.0 BE‐Basic Edition package (Lakeway Drive, College Station, TX, USA).

With the aim of finding a predictive model for optimal glycaemic control based on sociodemographic and clinical variables, we set out to establish a logistic regression model combining several of these variables. In order to establish a prediction model, it is strongly recommended to evaluate the model's performance using participant data other than those used for model development. Such external validation requires that, for each individual in the new dataset, outcome predictions are made using the original model (i.e., the calculated regression formula) and compared with the observed outcomes [21].

Accordingly, the initial sample was randomly divided into two parts (Samples A and B). One of these samples (Sample A—training set) was used to perform multiple logistic regression predictive models for the optimal glycaemic control variable through computational analysis of all possible equations combining clinical and sociodemographic variables, including age, sex, diabetes duration, BMI, HbA1c before sensor placement, CSII usage, daily insulin dose, smoking habits, diabetic retinopathy, diabetic nephropathy, years of sensor usage, and annual net income. First‐degree interactions of HbA1c before sensor placement with the other variables were analysed due to the clinical relevance of HbA1c as the most important variable related to T1D control.

Among all the models, the one with the best Akaike fit (Akaike's information criterion [AIC]) [22] and Schwarz's criterion (BIC) [23] was selected, provided that it met the Hosmer–Lemeshow's and Pearson's goodness‐of‐fit statistics. After selecting the best‐fitting model, non‐parametric receiver operating characteristic (ROC) curves were constructed for the maximum model, which included all variables and first‐degree interactions, and the selected model to compare their performance. Once the selected model was confirmed, influential values were examined using the Δ Beta method, which is analogous to Cook's distance in linear regression, and influential values were removed using the Hosmer, Taber, and Lemeshow method [24]. Validity indices were calculated for the selected model.

After model construction with Sample A, the model was subjected to validation in by two different methods. External validation was performed using the second part of the study sample as a validation cohort (Sample B—testing set), as described above. After obtaining the results, the difference between the two models (development cohort vs. validation cohort) was calculated to evaluate the estimated prediction loss of the validated model. The predictive loss (shrinkage [25]) of the model was estimated by the difference between the area under the ROC curve in the development cohort area under the curve (AUC) and the area under the ROC curve in the validation cohort AUC. Subsequently, internal validation was conducted using bootstrap resampling, as recommended by established guidelines [26, 27].

Finally, a risk calculator was developed based on the selected statistical model. The calculator used the regression formula to obtain a probability of glycaemic control in individual subjects based on their values of the variables included in the prediction model. A small sample of individuals from the validation cohort (Sample B) was used as a practical example to illustrate how the risk calculator would perform in real‐world settings.

## 3 Results

**FIGURE 1:**
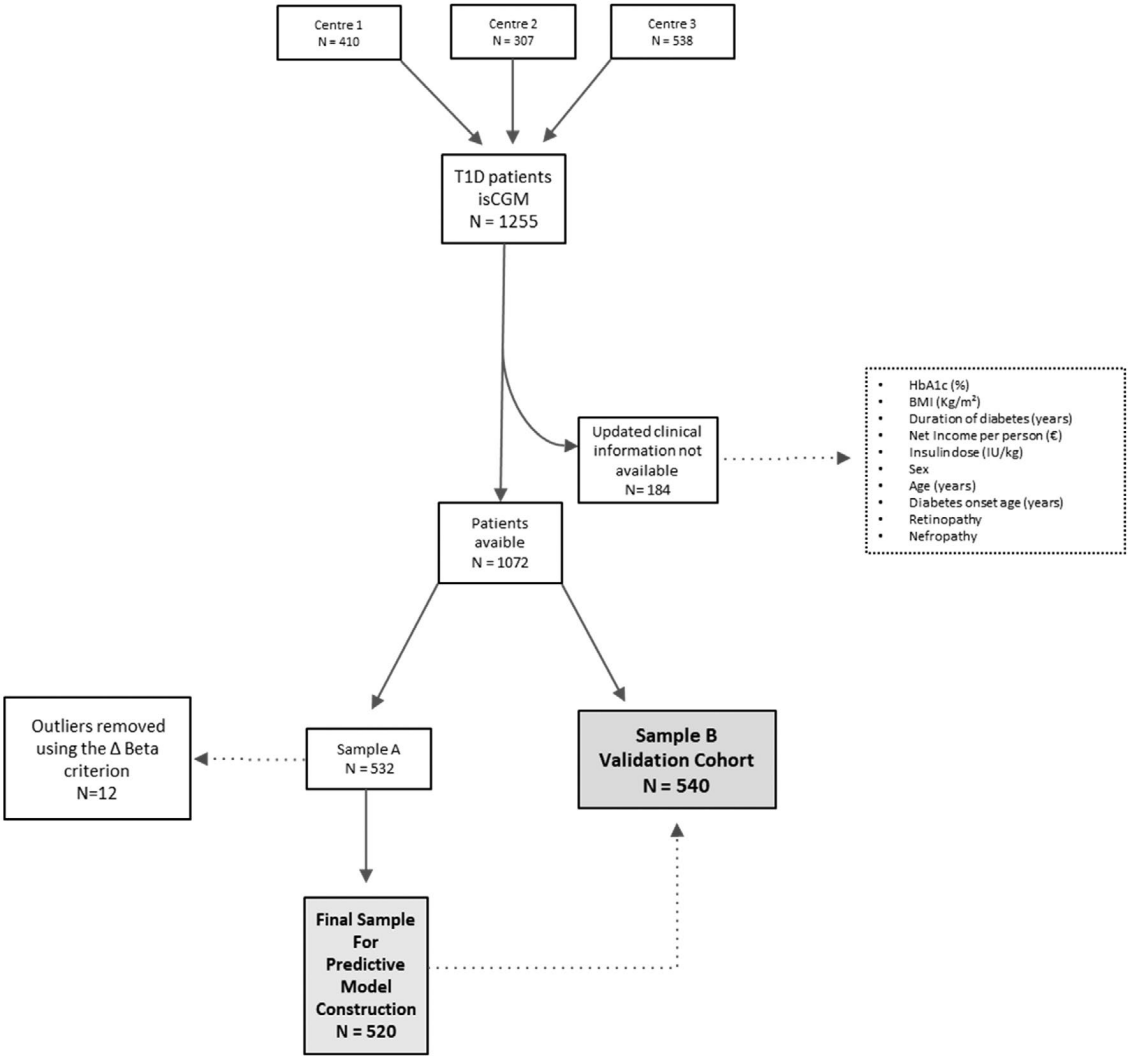
Study flowchart diagram. BMI, body mass index; HbA1c: glycated haemoglobin; isCGM, intermittently scanned continuous glucose monitoring; T1D, type 1 diabetes.

After applying the inclusion and exclusion criteria, 1072 individuals were selected for the study. The study flowchart diagram is shown in Figure 1. Mean age of this population was 47.8 (± 15.1) years, and 49% of the participants were female. Mean age at onset was 26.3 (± 15.7) years, and duration of diabetes was 21.5 (± 13.2) years. Mean HbA1c concentration prior to sensor placement was 62 ± 16 (7.8 ± 1.4) mmol/mol. Then, 20% of the individuals were active smokers, 25% showed diabetic retinopathy, and 12.5% exhibited diabetic nephropathy. Mean TIR was 61.5 ± 17.8, and mean TBR was 4.7 ± 4.8. The remaining baseline characteristics of the sample are presented in Table 1.

**TABLE 1:** Characteristics of the sample.

Variable	Obs *n* = 1072
Age	47.8 (± 15.1)
Sex, women	522 (49.0)
Diabetes onset age (years)	26.4 (± 15.7)
Net income/person/year (€)	16948.1 ± 5988.1
Multiple daily injections	1019 (95.1)
Insulin pump (CSII)	53 (4.9)
BMI (kg/m^2^)	25.9 (± 5.7)
Smokers	214 (20.0)
Duration of diabetes (years)	21.4 (± 13.3)
Pre‐isCGM HbA1c (mmol/mol, %)	62 ± 16 (7.8 ± 1.4)
Insulin (IU/kg)	0.60 ± 0.24
Retinopathy	268 (25.0)
Nephropathy^a^	123 (12.5)
TIR	61.4 ± 17.6
TBR < 70 mg/dL	4.6 ± 4.8
TAR > 180 mg/dL	34.0 ± 18.7
TAR > 250 mg/dL	11.4 ± 13.1
Coefficient of variation	36.7 ± 7.1

*Note:* Data are shown as mean ± standard deviation or number (%). TIR, time in range (70–180 mg/dL).

Abbreviations: BMI, body mass index; CSII, continuous subcutaneous insulin infusion; HbA1C, glycated haemoglobin; isCGM, intermittently scanned continuous glucose monitoring; TAR, time above range; TBR, time below range.

^a^ The available sample of individuals with nephropathy consisted of 1000 subjects.

**FIGURE 2:**
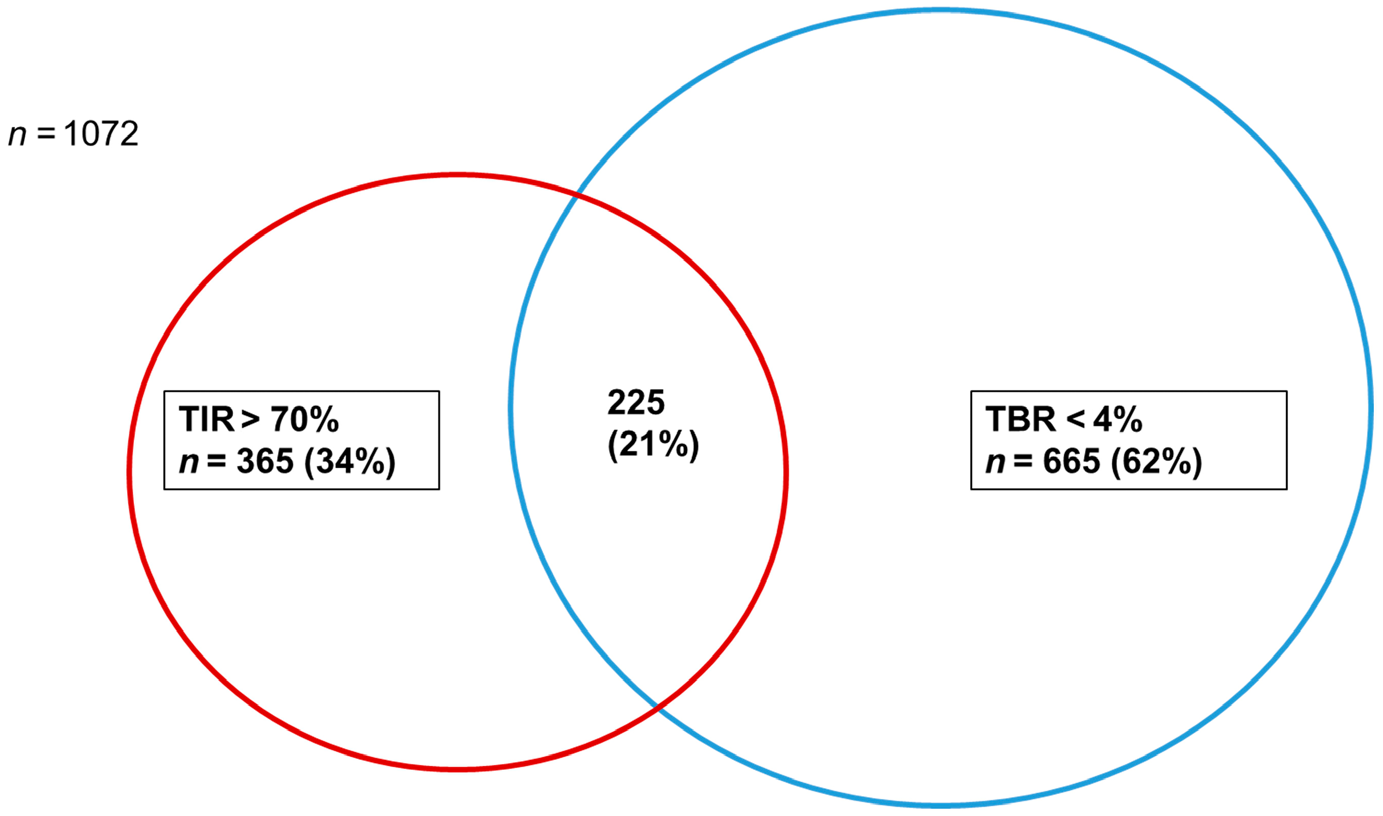
Venn diagram for optimal glycaemic control. The graph shows that 21% of the entire sample simultaneously shows TIR > 70% and TBR < 4%. TBR, time below range; TIR, time in range (70–180 mg/dL).

Three hundred sixty‐one (34%) individuals achieved the target of TIR > 70%, and 658 individuals (62%) had a TBR < 4%. Among these, 219 patients (21%) simultaneously had TIR > 70 mg/dL and TBR < 4% (Figure 2).

After randomly dividing the sample into two equal parts, Sample A (training set, 520 individuals) was used to construct predictive models for optimal glycaemic based on sociodemographic and clinical variables by logistic regression, and Sample B (testing set, 540 individuals) was used for validation. The characteristics of the randomly divided samples are displayed in Data S1 and S2. A total of 2314 possible hierarchical models were computationally analysed in the dataset used for model construction (Sample A). The best‐fitting model, determined by the AIC and Schwarz's criterion (BIC), included the variables age, sex, diabetes duration, prior HbA1c, annual net income per person, daily insulin dose, and the interaction of HbA1c with sex. The model exhibited good fit according to the Hosmer–Lemeshow statistics (*p* = 0.499) and the Pearson statistics (*p* = 0.209).

The maximum model, including all variables and interactions, was compared with the selected model. Both exhibited a high degree of statistical similarity (Data S3), with less than a 3% difference in the AUC and no statistically significant differences (*p* = 0.08).

The influential values of the selected statistical model were examined using the Δ Beta method, analogous to Cook's distance in linear regression. Subsequently, 12 influential values were removed using the Hosmer, Taber, and Lemeshow method [24] (Data S4).

**FIGURE 3:**
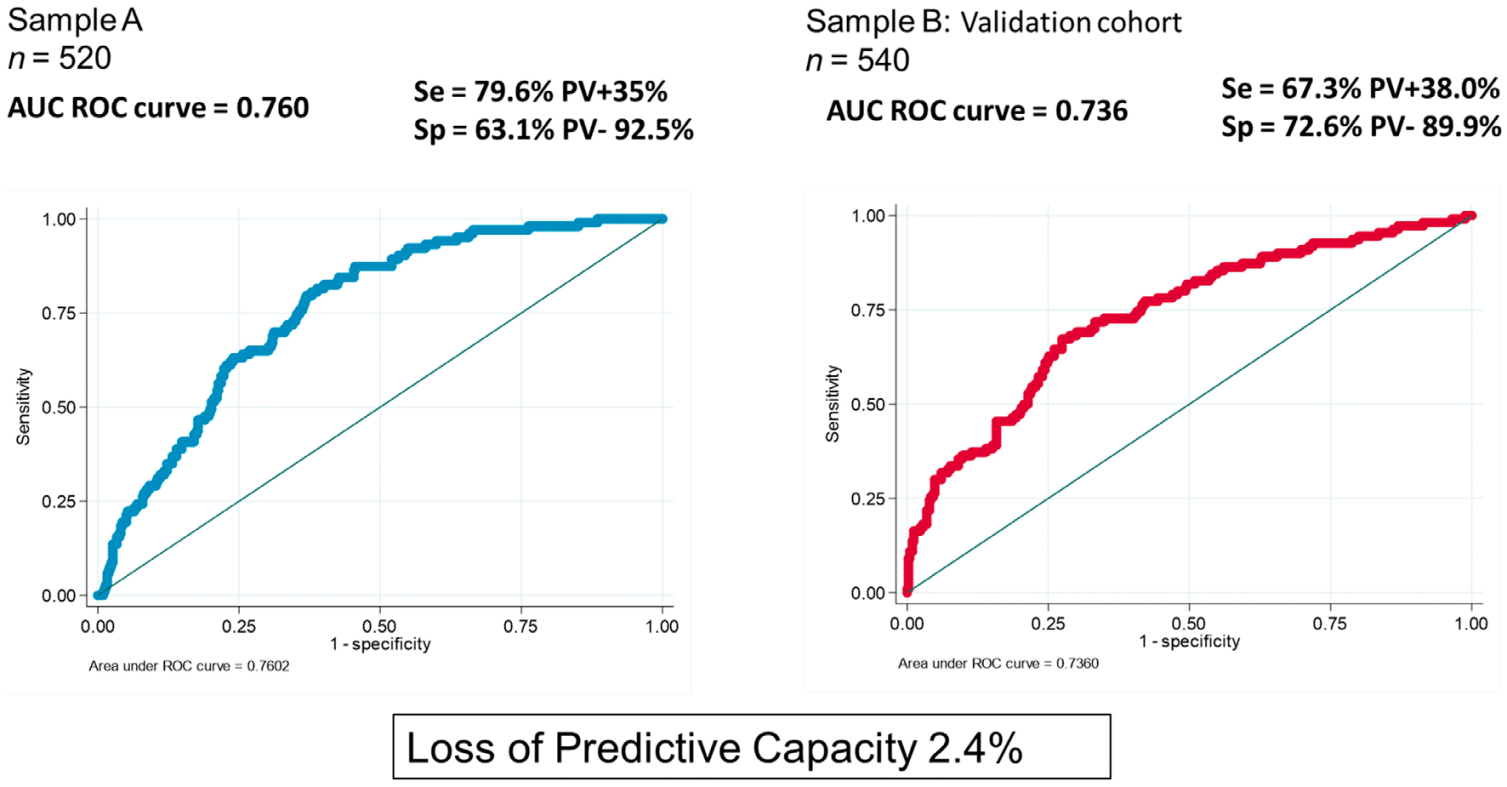
Validation of the selected model in an external validation cohort. The graph shows ROC curves for the selected model in the training cohort (Sample A) and the validation cohort (Sample B). External validation demonstrates the consistent predictive capability of the model when applied to the external validation cohort, with a model prediction loss < 5%. The overall model validity in the external cohort showed that the model accurately predicts the outcome in 80.4% of individuals. AUC, area under the curve; PV, predictive value; ROC, receiver operating characteristic; Se, sensitivity; Sp, specificity.

The model constructed using Sample A exhibited an AUC of 0.760, with a sensitivity of 79.6% and a specificity of 63.1% (Figure 3). Subsequently, after model construction, validation was conducted using Sample B as an external cohort. The model applied to the validation cohort demonstrated an AUC of 0.736, with a sensitivity of 67.3% and a specificity of 72.7%. The positive predictive value of the predictive model in the validation cohort was 38.0%, and the negative predictive value was 89.9% (Figure 3). The overall validity of the model, which assesses the agreement between predictions and actual outcomes, indicated that the model correctly classified 80.4% of the individuals.

Finally, a sensitivity test was performed through internal validation using Bootstrap resampling, which yielded an AUC of 0.756, closely resembling the results obtained through external validation.

**FIGURE 4:**
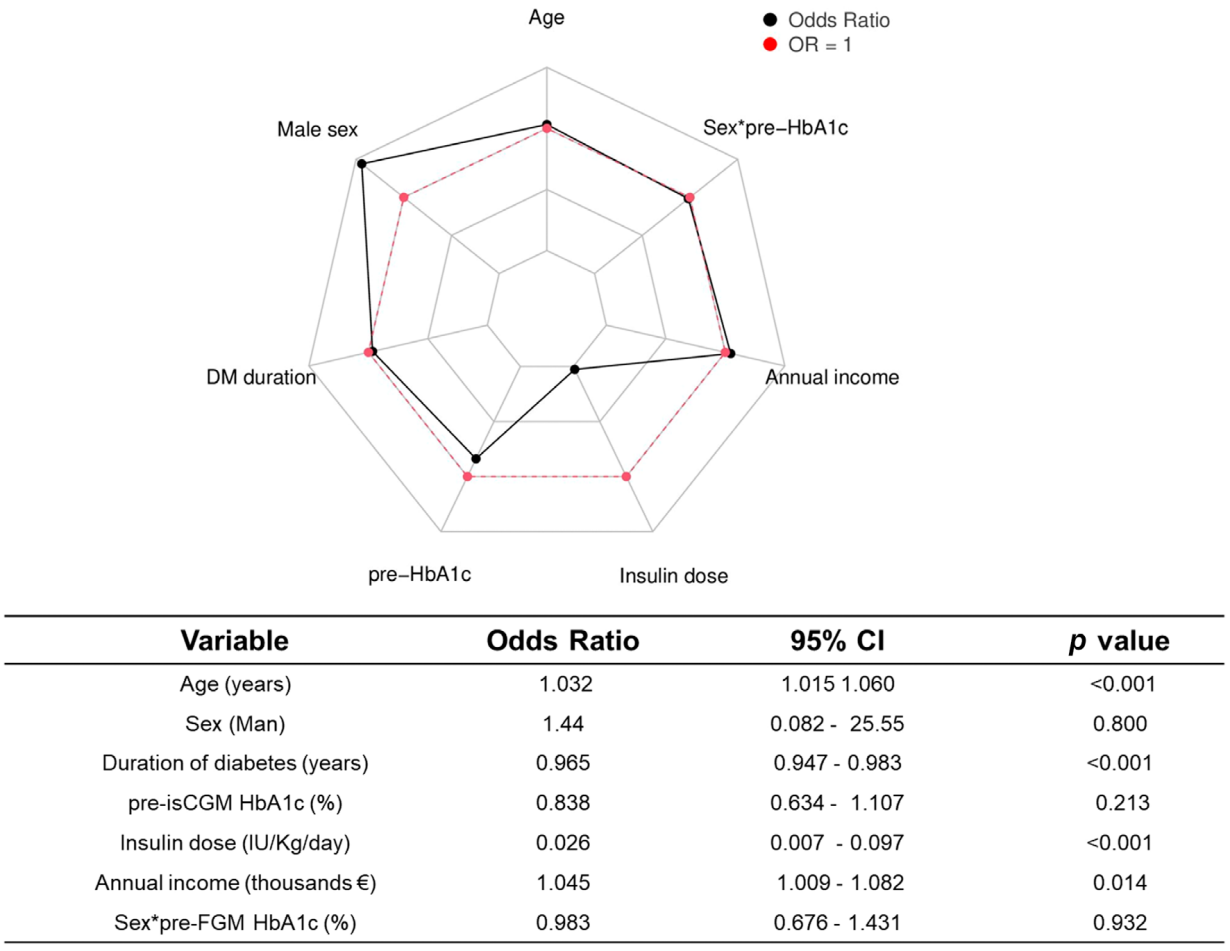
Risk values of the predictive variables included in the selected model applied to the validation cohort (Sample B). The table displays odds ratios for each of the variables included in the risk calculator for the validation cohort (Sample B). *, interaction; €, euros; CI, confidence interval; DM, diabetes mellitus; HbA1c, glycated haemoglobin; isCGM, intermittently scanned continuous glucose monitoring.

Based on the selected model, a risk calculator was developed that enables determining the probability of achieving glycaemic control in individual subjects from their values for the variables included in the model. The risk values obtained for all the variables after applying the calculator to the validation cohort are displayed in Figure 4.

To show how the calculator may be used, predictions were made for four individual cases of the validation cohort (Table 2). The examples illustrate cases where clinical characteristics and HbA1c levels were similar between patients, but predicted probabilities for achieving optimal control and actual glucose metrics differed. Cases 1 and 2 involved individual male patients aged 56 and 63, respectively, with diabetes duration exceeding 20 years and HbA1c levels within the target range before sensor placement. However, due to the influence of other characteristics included in the model, subject 1 had a predicted probability of achieving optimal control of 0.206 (95% CI: 0.04–0.59), much lower than that of subject 2, who had a value of 0.906 (95% CI: 0.64–0.98). The real results aligned reasonably well with the predictions, as subject 1 was far from optimal glycaemic control, while subject 2 was within the optimal range.

**TABLE 2:** Model predictions in individual subjects from the validation cohort.

Subject	Variables	Value	Predicted risk	Real TIR (70–180 mg/dL)	Real TBR < 70 mg/dL
1 (ID 902)	Sex	Man	0.206 95% CI (0.04–0.59)	62%	20%
	Age (years)	56			
	Duration of T1D (years)	24			
	Annual income (€)	11,863			
	Insulin doses/day (IU/kg)	1.25			
	HbA1c (mmol/mol)	48 (6.5%)			
2 (ID 809)	Sex	Man	0.906 95% CI (0.64–0.98)	73%	1%
	Age (years)	63			
	Duration of T1D (years)	20			
	Annual income (€)	32,183			
	Insulin doses/day (IU/kg)	0.33			
	HbA1c (mmol/mol)	52 (6.9%)			
3 (ID 453)	Sex	Woman	0.071 95% CI (0.05–0.10)	49%	1%
	Age (years)	44			
	Duration of T1D (years)	36			
	Annual income (€)	12,542			
	Insulin doses/day (IU/kg)	0.715			
	HbA1c (mmol/mol)	60 (7.6%)			
4 (ID 75)	Sex	Woman	0.630 95% CI (0.05–0.10)	87%	2%
	Age (years)	62			
	Duration of T1D (years)	11			
	Annual income (€)	25,536			
	Insulin doses/day (IU/kg)	0.18			
	HbA1c (mmol/mol)	60 (7.6%)			

*Note:* The table presents an example of predictions with the risk calculator in four subjects from the validation cohort (Sample B). The displayed information includes the variables used in the risk calculator, the predictions with confidence intervals, and, finally, the actual glucose metrics. This example shows how the risk calculator can easily provide an assessment of the probability of achieving optimal glycaemic control. In these patients, with similar values for certain variables at the time of CGM sensor placement, the prediction is reasonably consistent with the actual glucose metric values.

Abbreviations: CI, confidence interval; HbA1c, glycated haemoglobin; T1D, type 1 diabetes; TBR, time below range; TIR, time in range.

On the other hand, subjects 3 and 4 were both women with different clinical characteristics but with the same HbA1c levels at the time of isCGM device placement. Although both had HbA1c values that seemed to be far from the target control, subject 3 had a predicted probability of achieving optimal control of 0.071 (95% CI: 0.05–0.10), while subject 4 presented a much more favourable prediction of 0.630 (95% CI, 0.05–0.10). The actual glucose metrics data also aligned with the predictions in this case. The examples analysed above show that the risk calculator readily provides an assessment of the probability of achieving glycaemic control that could be useful in glycaemia management.

## 4 Discussion

This study develops statistical models through computational analysis based on clinically accessible variables at the time of isCGM sensor placement to predict the probability of achieving TIR > 70% with TBR < 4% during the follow‐up of individuals with T1D. Our work demonstrates the possibility of reasonably predicting the probability of achieving optimal glycaemic control assessed with an isCGM sensor with only five clinical variables and the SES.

CGM devices provide a wide range of glucose metrics that have contributed to obtain evidence on glycaemic control. TIR stands out as the most relevant glucose metrics among those offered by CGM devices [28]. TIR has shown a strong association with HbA1c levels [9], time spent in hyperglycaemia [10], and the likelihood of chronic complications [29]. Analysis of this parameter by both patients and healthcare professionals facilitates achieving glycaemic control in T1D [30]. The CV of glucose measures glycaemia variability in each individual, which is an important characteristic for evaluating the quality of T1D control [31]. Elevated CV values in CGM have been associated with increased risk of hypoglycaemia and poorer glucose metrics [32].

However, the analysis of hypoglycaemia is complex, as it represents a specific clinical context that cannot be solely assessed through glucose metrics. CGM systems contribute to reduce the frequency of hypoglycaemic events [33] and combined findings from clinical trials suggest that TBR < 70 mg/dL is a robust predictor of severe hypoglycemia [11]. The development of technological devices utilising support vector machine and support vector regression techniques has proven highly effective in predicting both nocturnal and daytime hypoglycaemia [34]. Moreover, as time spent above the target glucose range increases, TIR decreases, negatively impacting long‐term glycaemic control and increasing the risk of complications [9, 35], as previously discussed [10]. Current guidelines recommend maintaining TIR > 70% with TBR (< 70 mg/dL) < 4% [28]. These target glucose metrics for glycaemic control have already been adopted as an endpoint in diabetes studies [14, 36, 37, 38] and therefore show potential as a valuable outcome measure for assessing T1D in CGM users.

The use of predictive models to explore personalised approaches for disease management is not new in endocrinology. In other areas within this field, models have already been developed to validate treatment selection algorithm for SGLT2 inhibitor and DPP‐4 inhibitor therapies in type 2 diabetes [39] However, the use of predictive models based on various clinical and socio‐economic variables for studying optimal glycaemic control in diabetes, assessed by isCGM glucose metrics, remains unexplored.

While the determinants of glycaemic control are multiple and diverse, many studies only focus on comparing the impact of technology use, rather than delving into the effect of various variables involved in the chronic control of individuals with T1D.

To create a predictive statistical model through computational methods we used a multicentre cohort where all subjects were users of isCGM based on the same isCGM system, used this system appropriately, and were assessed at the same time. We included variables involved in the chronic control of individuals with T1D, such as SES [14], and smoking [40]. These variables have gained significance in the last decade and are currently considered factors consistently associated with glycaemic control, complications, and even mortality in T1D [41]. We found that our model could predict the probability of optimal glycaemic control reasonably well in future CGM users, since the model was validated with robust results in an external validation cohort and through internal validation by bootstrap resampling, as recommended [26]. The model achieved an AUC close to 0.75. An additional advantage of our model is its easy application, as it is based on variables readily accessible and widely used in clinical practice. The model provides a parameter, probability, with high informative value that enables additional insights to less experienced clinicians into how to improve their decision‐making process. Furthermore, the model's high negative predictive value, close to 90%, could facilitate the identification of individuals who are likely to fall outside the recommended glycaemic control, enabling the development of tailored therapeutic strategies for them. This individualised strategy could take us one step closer to precision medicine [42, 43], through individual assessment of treatment effectiveness.

This study has some limitations. First, the patient sample used for the construction and validation of the model was obtained from a retrospective cohort and therefore the variables could not be controlled. Second, diabetes control was assessed in a binary manner. Although this approach is widely used, it simplifies a complex clinical reality. Diabetes control is better represented as a continuum, in which glucose levels vary significantly among patients. By reducing this variability to a single threshold, there is a risk of oversimplifying the evaluation of glycaemic control, potentially leading to inaccuracies. This binary approach also fails to capture gradual changes in clinically relevant outcomes, such as gradual improvements or deteriorations in glycaemic control. Third, variables associated with a higher risk of hypoglycaemia, such as the Clarke test, physical activity, or alcohol consumption [44, 45], were not included as they were not systematically available in the medical records. Finally, some significant variables that could influence chronic control, such as C‐peptide, and diet, could not be used to construct the model because they were also absent from the medical records.

## 5 Conclusion

Clinical and socio‐economic characteristics reasonably predict the probability of achieving TIR > 70% with TBR < 4% in T1D. Statistical models based on these variables could help to identify patients with risk phenotypes, thereby contributing to tailor decisions to their needs, and optimise available resources leading to a more personalised medicine. Prospective studies will be required to further validate the predictive power of clinical and socio‐economic variables for the chronic control of T1D and its complications.

## Author Contributions

**Fernando Sebastian‐Valles:** conceptualisation, methodology, supervision, writing – original draft. **Jose Alfonso Arranz Martin:** conceptualisation, investigation, writing – review and editing. **Julia Martínez‐Alfonso:** data curation, formal analysis, writing – review and editing. **Jessica Jiménez‐Díaz:** data curation, investigation. **Iñigo Hernando Alday:** resources, investigation. **Victor Navas Moreno:** investigation, visualisation. **Teresa Armenta Joya:** methodology, investigation. **Maria del Mar Fandiño García:** resources, data curation. **Gisela Liz Román Gómez:** data curation, software. **Jon Garai Hierro:** resources, investigation. **Luis Eduardo Lander Lobariñas:** writing – review and editing, visualisation. **Carmen González‐Ávila:** formal analysis, writing – review and editing. **Purificación Martinez de Icaya:** investigation, project administration. **Vicente Martínez‐Vizcaíno:** supervision, funding acquisition. **Mónica Marazuela:** supervision, methodology, writing – review and editing funding acquisition. **Miguel Antonio Sampedro‐Nuñez:** conceptualisation, methodology, supervision, writing – review and editing, funding acquisition.

## Ethics Statement

The Research Ethics Committee of Hospital de La Princesa, Madrid, (Study number: 5084‐01/2023) approved this study and waived informed consent from patients. The research was conducted according to the principles of the Declaration of Helsinki.

## Consent

The authors have nothing to report.

## Conflicts of Interest

The authors declare no conflicts of interest.

## Supporting information


Data S1.

## Data Availability

The datasets used and/or analysed during the current study are available from the corresponding author on reasonable request.
