# Supplementary material for: Predicting Time in Range Without Hypoglycaemia Using a Risk Calculator for Intermittently Scanned CGM in Type 1 Diabetes
Source: Endocrinol Diabetes Metab. 2024 Dec 24;8(1):e70020. doi: 10.1002/edm2.70020 (PMC11667215; doi:10.1002/edm2.70020)

Supplementary S1

Variable	Sample A<br>*n= 532*	Sample B<br>*n= 540*	P value
Age	47.4 ±15.1	48.1 ±15.2	0.468
Sex women	263 (49.4)	261 (48.40)	0.752
Diabetes onset age (years)	25.9 ±15.4	26.4 ±16.0	0.582
Net income/person/year (€)	16850 ± 5768.8	17063 ± 6197.3	0.554
Multiple daily injections	503 (94.6)	518 (96.2)	0.201
Insulin pump (CSII)	29 (5.4)	32 (3.8)	0.201
BMI (Kg/m^2)	25.7 (± 4.5)	25.8 (± 6.5)	0.853
Smokers	102 (19.1)	116 (21.5)	0.332
Duration of diabetes (years)	21.3 (± 12.5)	21.5 (± 14)	0.894
Mean pre-FGM HbA1c (mmol/mol)	7.9 ± 1.4 (63±15)	7.9 ± 1.5 (63±16)	0.559
Insulin (IU/Kg)	0.61 ± 0.24	0.59 ± 0.24	0.088
Retinopathy	126 (23.6)	142 (26.3)	0.286
Nefropathy[a]	59 (11.8)	66 (13.07)	0.542

Supplementary S2

Variable	Sample A<br>*Obs*<br>*n =532*	Sample B<br>*Obs*<br>*n =540*	P value
TIR	62.0 ± 16.7	60.9± 18.4	0.274
TBR<70 mg/dL	4.7 ± 4.7	4.5 ± 5.0	0.373
TAR>180 mg/dL	33.3 ± 17.7	34.7 ± 19.6	0.207
TAR >250 mg/dL	10.7 ± 12.2	12.1 ± 14.0	0.089
Coefficient of Variation	36.8 ± 7.1	36.5 ± 7.2	0.484

Supplementary S3

Comparison between ROC curves for the Maximum Model and the Selected Model

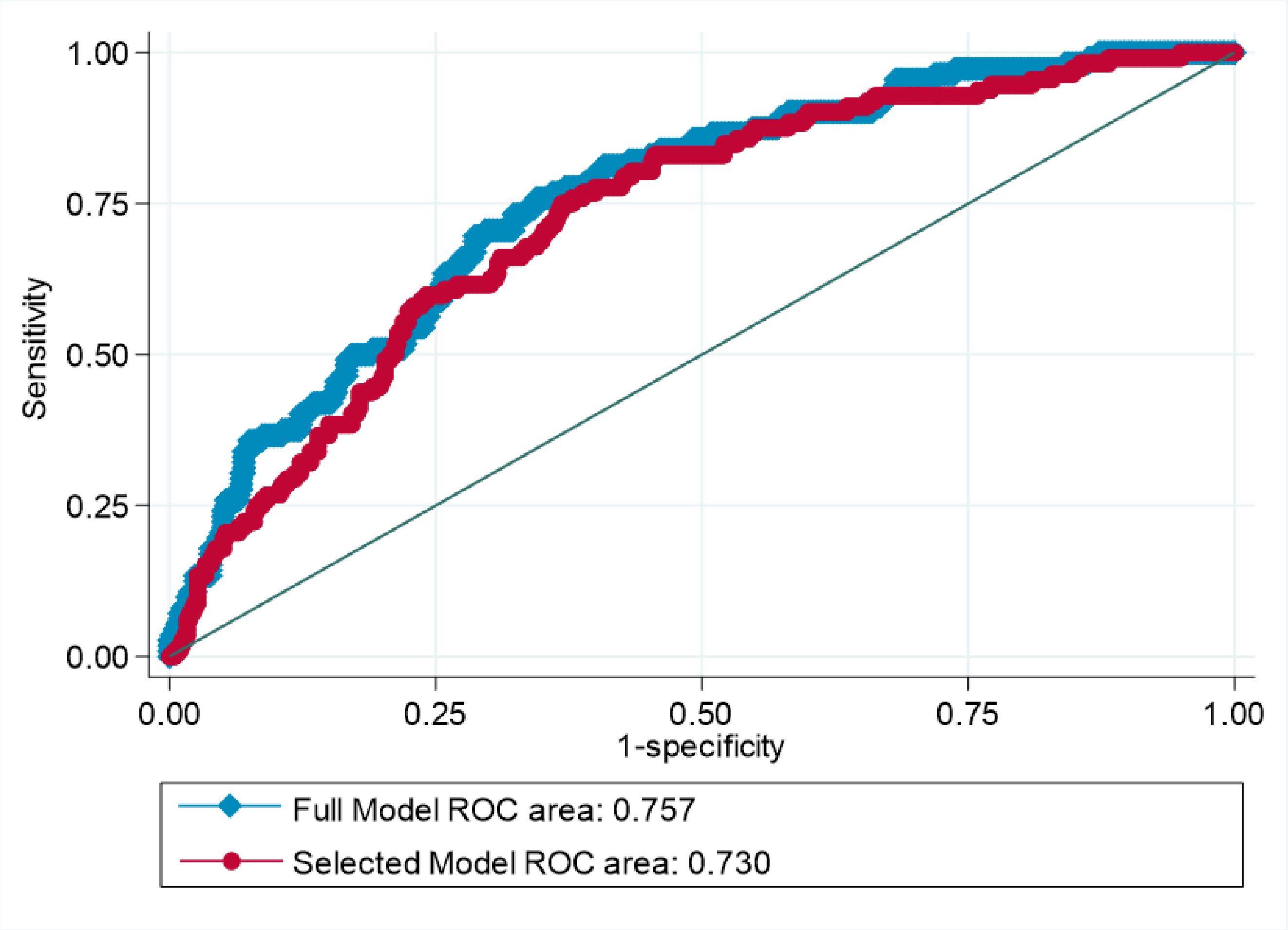

Comparison of the Area Under the Curve (AUC) of the receiver operating characteristic curve (ROC) between the maximal model, which includes all variables and interactions, and the selected model, which only includes age, diabetes duration, prior HbA1c, annual net income per person, daily insulin dose, and the interaction of HbA1c with age. A significant similarity is observed between the selected model and the maximal model, demonstrating the competency of the selected model.

Supplementary S4

Δ Beta

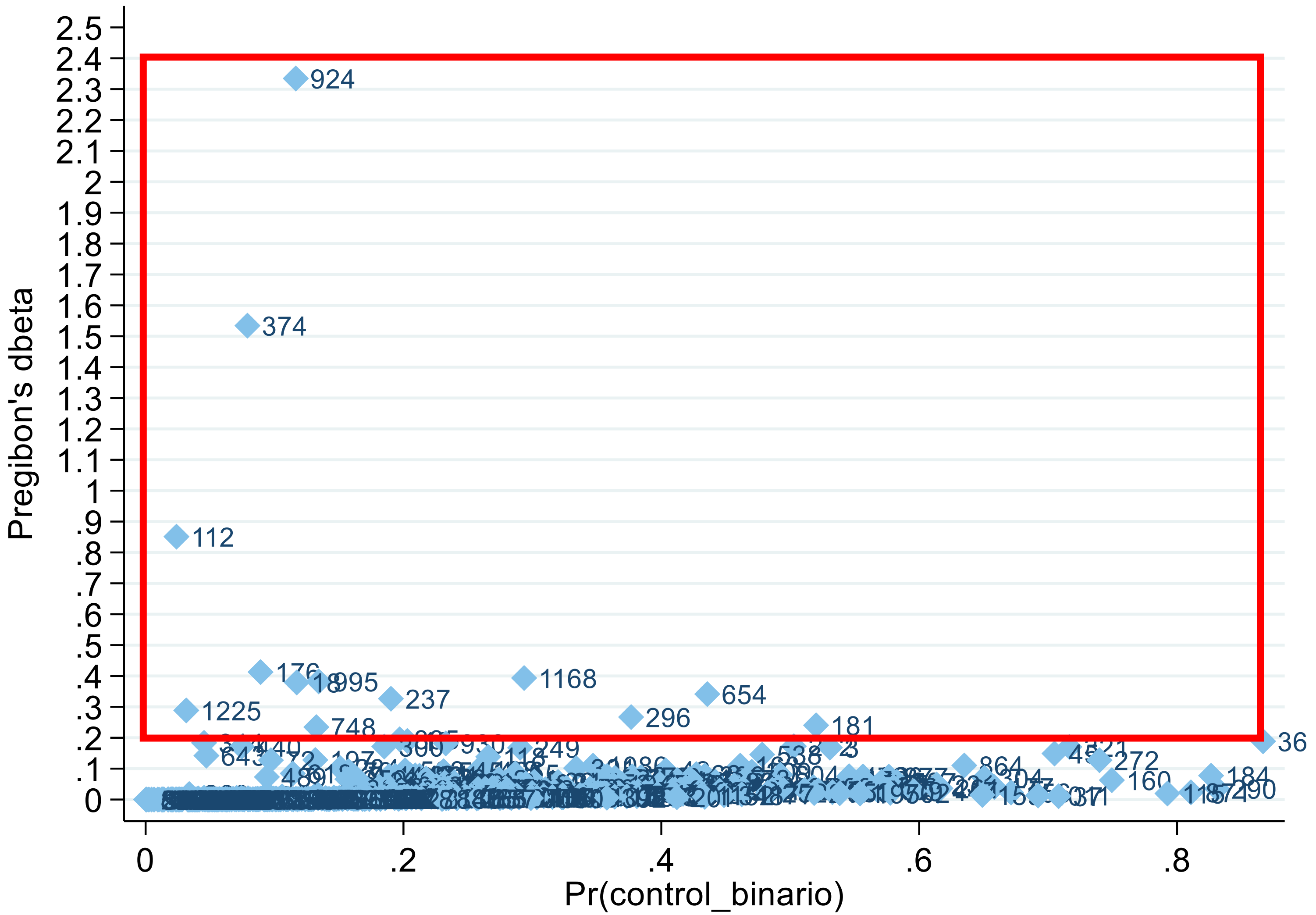

Supplement: Supplementary file 1 — Data S1. [file EDM2-8-e70020-s001.pdf]
